# Supplementary material for: Clinical Characterization of Alagille Syndrome in Patients with Cholestatic Liver Disease
Source: Int J Mol Sci. 2023 Jul 21;24(14):11758. doi: 10.3390/ijms241411758 (PMC10380973; doi:10.3390/ijms241411758)

Sanger sequencing results

Case 1. c.1619_1622dup (p.(Tyr541Ter))

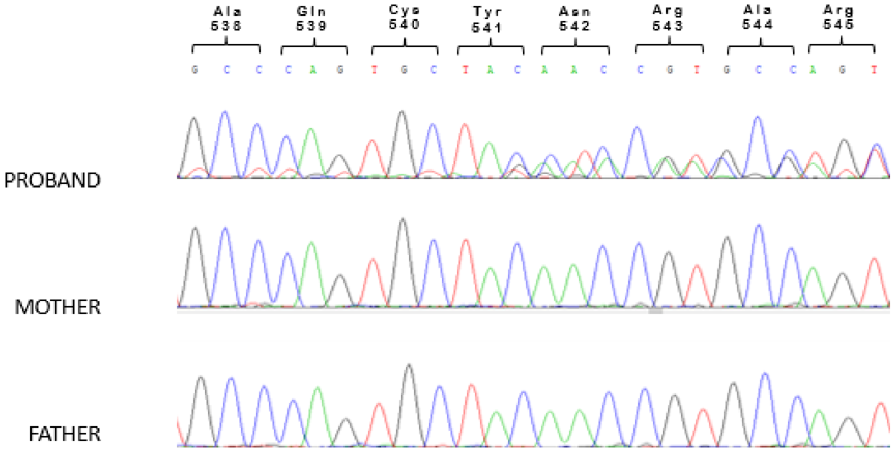

Case 2. c.1160del (p.(Gly387AlafsTer25))

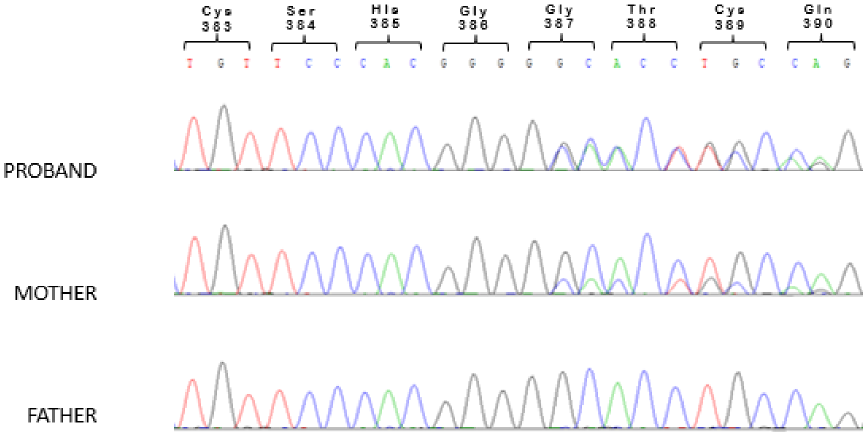

Case 3. c.2122_2125del (p.(Gln708ValfsTer34))

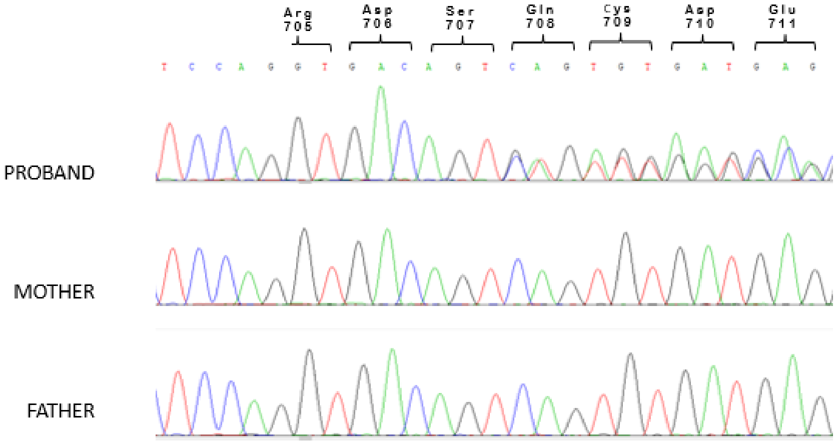

Case 4. c.1976G>A (p.(Trp659Ter))

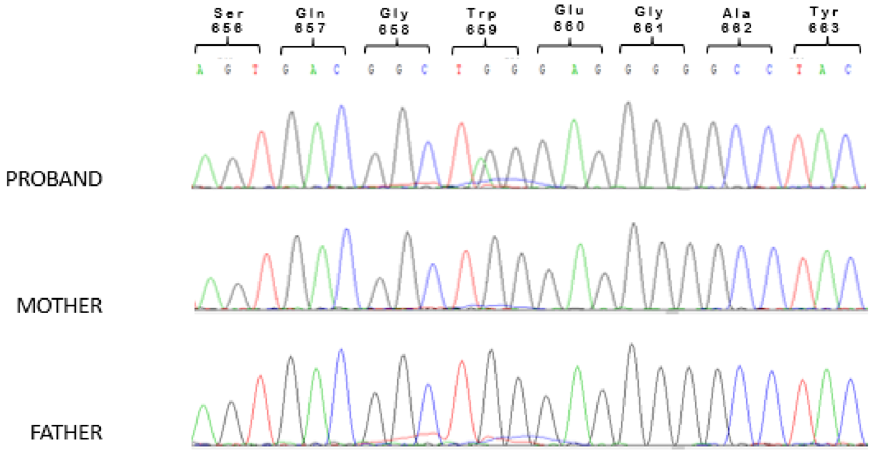

Case 5. c.964dup (p.(Cys322LeufsTer5))

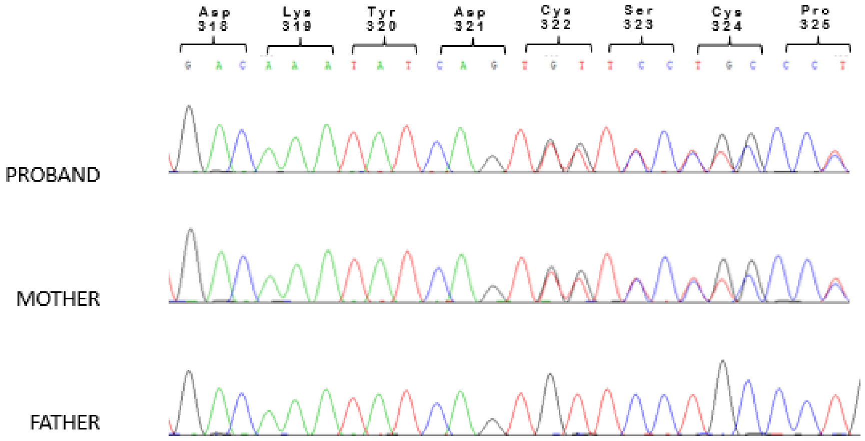

Case 6. c.1395+1G>A

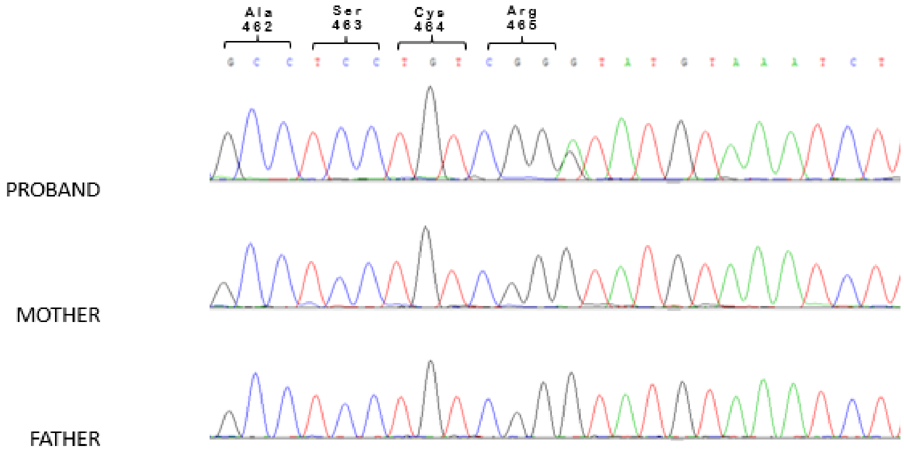

Case 8 and Case 9. c.1348+1G>A

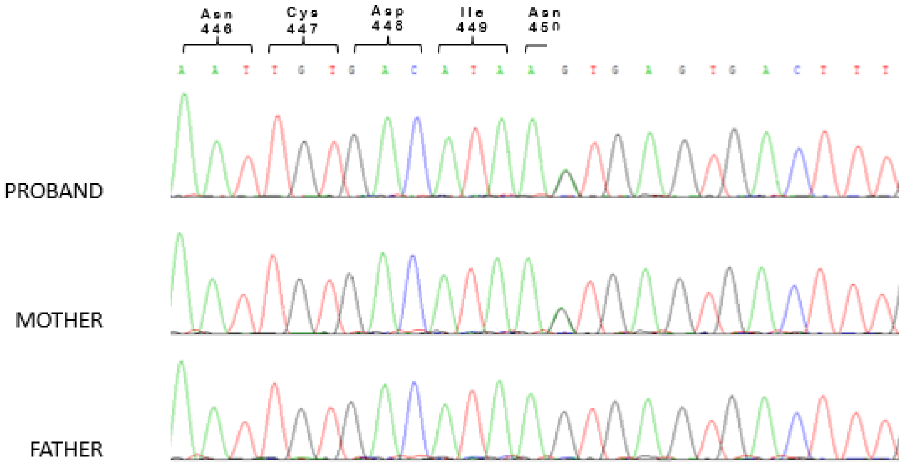

Case 11. c.2806T>G p.Cys936Gly

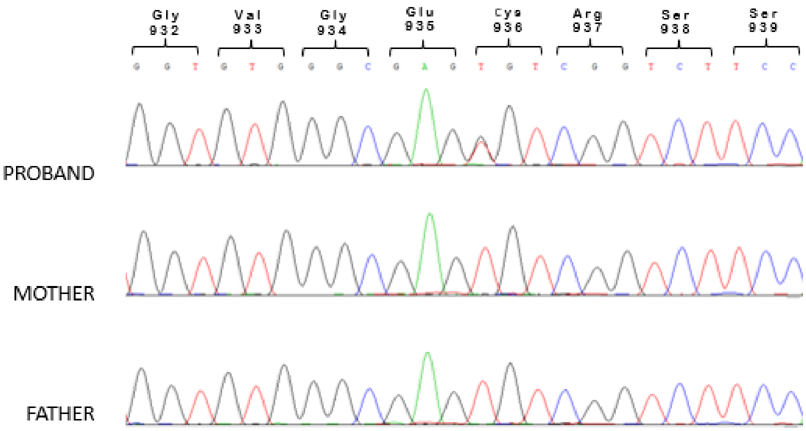

Case 13. c.3168_3169del (p.(Arg1056SerfsTer52))

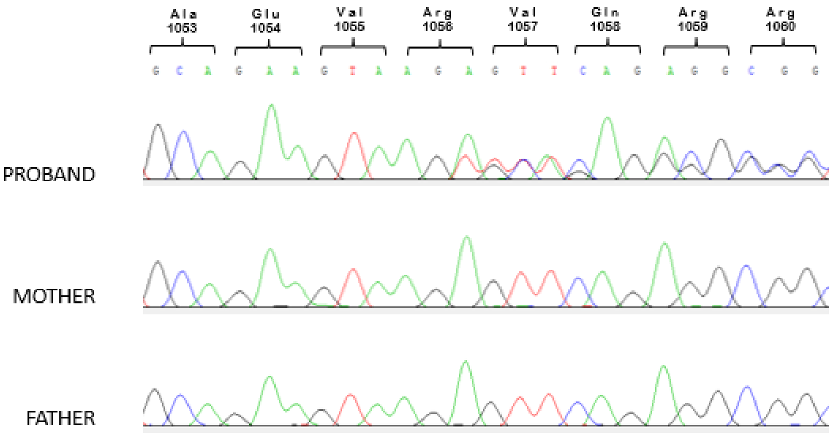

Case 15. c.695-2A>G

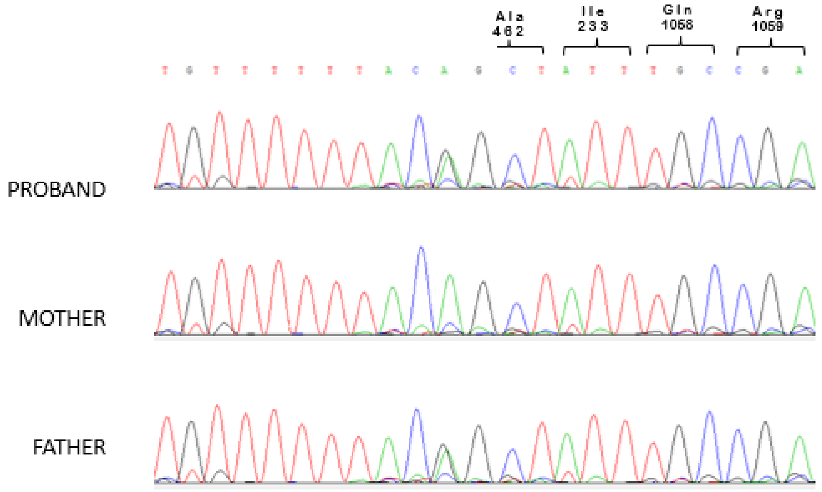

Case 17. c.164dup (p.(Cys55TrpfsTer18))

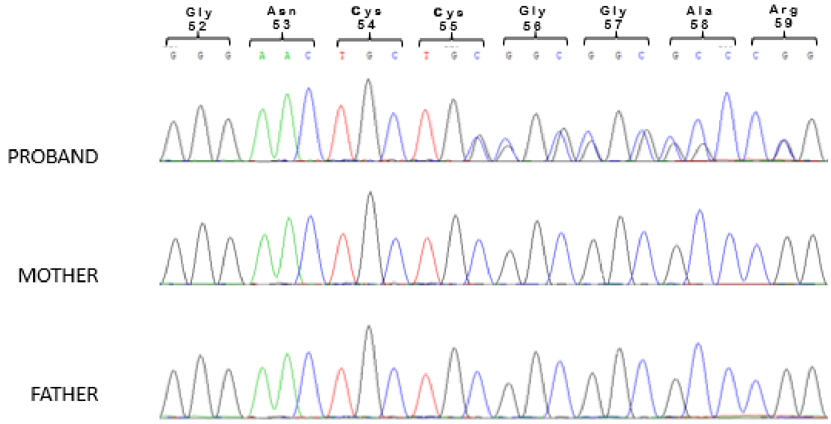

18. c.439+1G>A

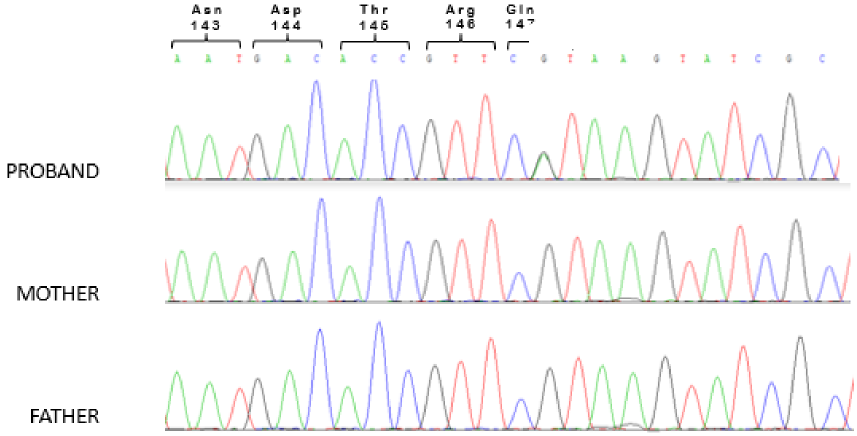

Supplement: Supplementary file 1 [file ijms-24-11758-s001.zip › Sanger sequencing results S2.pdf]
