# Supplementary material for: Clinical Characterization of Alagille Syndrome in Patients with Cholestatic Liver Disease
Source: Int J Mol Sci. 2023 Jul 21;24(14):11758. doi: 10.3390/ijms241411758 (PMC10380973; doi:10.3390/ijms241411758)
Supplement: Supplementary file 1 [file ijms-24-11758-s001.zip › Table S1_AM_.pdf]

| Patients | hg19 | NM_000214.3 | NP_000205.1 | gnomAD v2.1.1 global MAF | MetaDome* | CADD phred | Eigen phred | MPA score** | SPiP | spliceAI max score† | SIFT*** | HumDiv**** | Polyphen 2 HumVar***** | Fathmm****** | REVEL******* | ClinPred******* | Meta SVM******** | Meta LR********* | Mistic********** | ACMG | References |
|---|---|---|---|---|---|---|---|---|---|---|---|---|---|---|---|---|---|---|---|---|---|
| 10 | chr20:g.10622218A>C | c.2806T>G | p.(Cys936Gly) | n/a | 0,58 | 25 | 6,84 | 9,0 | No effect on splicing | 0 | 0,004 | 0,6 | 0,3 | -3,81 | 0,902 | 1,000 | 1.0071 (10) | 0.9001 (10) | 0,94 | LPat (PM2, PP3, PS2) | novel |

* The closer to 0, the more intolerant to variation
** Raw score [0;10], 10: high impact
† Thresholds ≥ 0.2|0.5|0.8 for impact
*** Threshold < 0.05 for Damaging
**** Thresholds ≥ 0.454|0.957 for Possibly and Probably Damaging
***** Thresholds ≥ 0.447|0.909 for Possibly and Probably Damaging
****** Threshold ≤ -1.5 for Damaging
****** Thresholds 0.2|0.5 for Benign, Uncertain, Damaging
******* Threshold ≥ 0.5 for Damaging
******** Threshold ≥ 0 for Damaging (reliabilty index: 0-10), 10:high
********* Threshold ≥ 0.5 for Damaging (reliabilty index: 0-10), 10:high
********** Threshold ≥ 0.5 for Damaging
